# Choroidal Thickness Increases During Parasympathetic Dominance After Immersion of the Foot in Warm Water

**DOI:** 10.7759/cureus.53194

**Published:** 2024-01-29

**Authors:** Natsumi Toh, Yuki Hashimoto, Fuka Kuwahara, Miki Yoshimura, Sakurako Imabayashi, Takeshi Yoshitomi

**Affiliations:** 1 Department of Orthoptics, Fukuoka International University of Health and Welfare, Fukuoka, JPN

**Keywords:** systemic circulation, subfoveal choroidal thickness, parasympathetic activity, enhanced depth imaging optical coherence tomography, choroidal morphology

## Abstract

Purpose: This study aimed to assess the course of changes in choroidal morphology after immersion of the foot in warm water at 40°C using enhanced depth imaging optical coherence tomography (EDI-OCT).

Methods: Forty-three right eyes of 43 healthy participants were included. Changes in choroidal morphology were determined using EDI-OCT to evaluate subfoveal choroidal thickness (SCT). Systolic, diastolic, and mean blood pressures (SBP, DBP, and MBP, respectively) were also measured to determine systemic circulatory dynamics at baseline, immediately after immersion (0 min), and 10, 20, and 30 min after immersion.

Results: Immediately after immersion, SBP, DBP, and MBP were significantly declined versus baseline. In contrast, the SCT was significantly increased after warm water immersion. However, all these parameters did not change significantly compared to the baseline within 30 min.

Conclusion: In the normal eye, parasympathetic nerve activity induced by warming stimuli increases choroidal morphology in response to a decrease in systemic circulatory activity, which normalizes within 30 min. The findings of this study may provide basic data for the prevention and treatment of various choroidal diseases in which sympathetic hyperactivity is involved in the pathogenesis.

## Introduction

Choroidal vessels are directly affected by autonomic and systemic circulatory dynamics because of their weak autoregulatory capacity [1,2]. Increased sympathetic activity leads to vasoconstriction and a rise in blood pressure (BP), whereas increased parasympathetic activity induces vasodilation, resulting in a decrease in BP. Regarding choroidal changes during autonomic nervous activity, in the wintertime, during the day, in the mid-luteal phase, and after cold stimulation as a stress test, choroidal circulations increase, and choroidal morphology decreases when sympathetic activity is predominant [3-8]. However, in the summertime, during the night, and in the late follicular phase, when parasympathetic activity is dominant, choroidal circulations are decreased, and choroidal morphology is increased [3-5,8,9].

Several reports exist on thermal stimulation of the feet [9-12]. The temperature of the nasal mucosa increases after immersion of the feet in warm water at 42°C, suggesting a decrease in sympathetic activity and an increase in parasympathetic activity in the nasal vascular system [10]. Furthermore, foot bathing at 42°C in young healthy Japanese adults increases parasympathetic activity and decreases sympathetic activity [11]. A recent report showed that immersion in 40°C warm water induces a predominance of parasympathetic activity and a decrease in systemic circulation, resulting in a simultaneous decrease in choroidal blood flow velocity at the macula, and revealed a significant positive correlation between choroidal blood flow velocity and systemic circulation dynamics [9]. However, no studies have evaluated choroidal morphology following warm water immersion to date.

Retinochoroidal diseases, such as central serous chorioretinopathy (CSC), involve sympathetic hyperactivity in their pathogenesis and changes in choroid [13,14]. Therefore, providing fundamental data to assess how increased parasympathetic activity resulting from warm water immersion affects choroidal morphology is necessary. This evaluation may help determine its potential to prevent or alleviate retinochoroidal disease development and symptoms. Thus, this study aimed to investigate the course of choroidal morphological changes after immersion in warm water at 40°C using enhanced depth imaging optical coherence tomography (EDI-OCT).

## Materials and methods

Participants

This study adhered to the tenets of the Declaration of Helsinki, and all participants provided written informed consent. Moreover, the study protocol was approved by the Ethics Committee of Fukuoka International University of Health and Welfare (approval ID: 20-fiu hw-022). This study was a prospective study conducted at Fukuoka International University of Health and Welfare in Fukuoka, Japan, and included 43 right eyes of 43 healthy young Japanese adults. The number of participants in this study is based on previous reports describing choroidal morphology and circulatory dynamics related to cold and warm stimuli affecting the autonomic nervous system [6,7,9]. These volunteers were all recruited through a non-probability sampling with voluntary responses. All participants enrolled showed best-corrected visual acuity (BCVA) of 20/20 or better and no abnormalities in fundus findings, had no cardiovascular or ophthalmologic disease, and were not using ophthalmic or systemic medications. Each individual was assessed for BCVA, color fundus photography, intraocular pressure (IOP), BP, heart rate (HR), and EDI-OCT.

Warm water immersion

The tests were conducted at a room temperature of 24°C and a humidity level of 47%. For immersion in warm water, both feet were immersed up to the ankle at 40°C for 60 s [9]. All examinations were taken at baseline, immediately after immersion (0 min), and 10, 20, and 30 min after immersion. Participants were instructed to refrain from smoking or exercising for at least two hours before testing. At all measurement points, BP and HR were measured first, and then EDI-OCT was performed second, subsequently followed by IOP testing.

IOP and systemic hemodynamics measurements

IOP, BP, and HR were measured at baseline, immediately after immersion, and 10, 20, and 30 min after immersion, respectively. Mean blood pressure (MBP) was calculated from systolic blood pressure (SBP) and diastolic blood pressure (DBP) according to the following equation: $MBP=DBP+\frac{1}{3}(SBP-DBP)$.

EDI-OCT measurements

**Figure 1:**
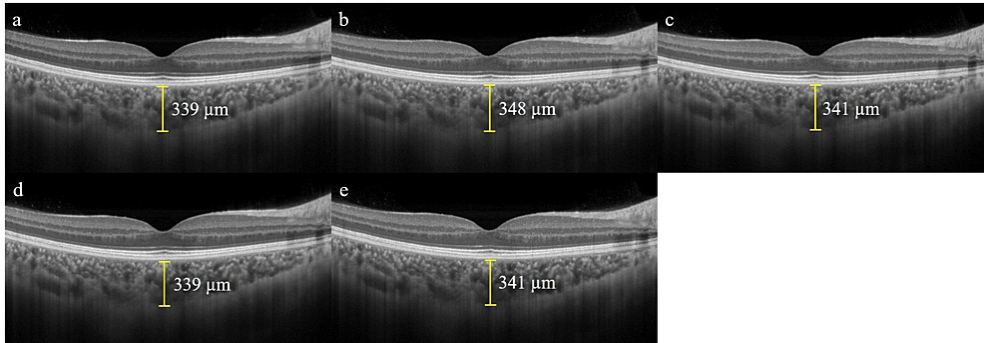
Enhanced depth imaging optical coherence tomography images at baseline (a), immediately after the immersion (b), and at 10 min (c), 20 min (d), and 30 min (e) later in a participant (case 6). SCT was 339 μm at baseline (a), 348 μm at 0 min (b), 341 μm at 10 min (c), 339 μm at 20 min (d), and 341 μm at 30 min (e), respectively. SCT was thickest after warm water immersion. SCT: subfoveal choroidal thickness

The fovea was scanned horizontally using EDI-OCT (RS-3000 Advance 2; NIDEK, Gamagori, Japan), and two examiners (YH and MY) manually measured the vertical distance between the outer edge of the high reflectance line corresponding to the retinal pigment epithelium and the choroid in a participant information masked condition (Figure 1). The average subfoveal choroidal thickness (SCT) measured by the two examiners was calculated. To obtain high-quality images for analysis, the images were taken in superfine mode with 120 additions of B-scan images and a signal strength index of more than 8. In addition, EDI-OCT was examined using a follow-up function that took images of the same area as the baseline.

Statistical analysis

Statistical analysis included Friedman's test and Scheffe's paired comparison test to examine variations in IOP, SBP, DBP, HR, MBP, and SCT among the five measurement points (baseline and 0, 10, 20, and 30 min). Spearman's rank correlation coefficient was used to examine the correlations of the SCT changes with refractive error and axial length. Statistical significance was set at P<0.05.

## Results

Forty-three eyes of 43 healthy volunteers (25 women and 18 men) were examined in this study. The mean age was 22.1±4.7 years (range, 18-39 years). The mean refractive error was −3.1±3.3 D, ranging from +1.6 to −11.3 D, and the mean axial length was 24.7±1.4 mm, ranging from 21.1 to 27.2 mm.

Ocular and systemic hemodynamics data

IOP, SBP, DBP, HR, and MBP changes are summarized in Table 1. Friedman's test on SBP, DBP, MBP, and IOP showed significant changes (P<0.001, P<0.001, P<0.001, and 0.026, respectively). SBP, DBP, and MBP were significantly reduced from baseline immediately after warm water immersion (P<0.001, P<0.001, and P<0.001, respectively), whereas they were no longer significantly different from baseline within 30 min. Friedman's test for HR revealed no significant changes (P=0.451).

**Table 1:** Changes in ocular biometric parameters and systemic factors at baseline and after the warm water immersion. Values are presented as mean±SD. SD: standard deviation; min: minutes; IOP: intraocular pressure; SBP: systolic blood pressure; DBP: diastolic blood pressure; MBP: mean blood pressure; HR: heart rate; bpm: beats per minute; SCT: subfoveal choroidal thickness ***P<0.001, **P<0.01, *P<0.05

		After the warm water immersion				Friedman's test (P-value)	Scheffe's paired comparison (P-value)			
	Baseline	0 min	10 min	20 min	30 min		0 min	10 min	20 min	30 min
IOP (mmHg)	13.6±2.5	14.0±2.7	13.6±2.6	13.4±2.6	13.7±2.7	0.026	0.412	0.988	0.802	0.998
SBP (mmHg)	110.3±11.2	104.6±12.3***	106.4±12.1*	107.2±11.5	107.5±11.6	<0.001	<0.001	0.010	0.116	0.056
DBP (mmHg)	69.6±7.6	65.3±9.1***	67.8±7.8	67.9±8.6	69.0±7.3	<0.001	<0.001	0.062	0.167	0.936
MBP (mmHg)	83.2±8.3	78.4±9.6***	80.7±8.6**	81.0±9.0	81.8±8.2	<0.001	<0.001	0.008	0.118	0.487
HR (bpm)	80.5±11.9	79.9±10.9	79.1±10.4	78.9±11.2	79.7±11.1	0.451	0.982	0.759	0.545	0.957
SCT (µm)	271.4±77.0	277.2±78.1***	274.6±77.9	273.6±77.1	272.9±76.4	<0.001	<0.001	0.122	0.603	0.623

EDI-OCT data

The SCT changes are shown in Table 1 and Figure 1. There was no significant difference in the SCT measurements of the two examiners (P=0.733). The average SCT values at baseline, immediately after warm water immersion (0 min), and 10, 20, and 30 min after warm water immersion were 271.4±77 µm, 277.2±78.1 µm, 274.6±77.9 µm, 273.6±77.1 µm, and 272.9±76.4 µm, respectively. Friedman's test on SCT showed significant changes (P<0.001). Moreover, the mean SCT values increased significantly by +5.8 µm at 0 min (P<0.001), with no significant differences observed from baseline at 10 min or later (Table 1). There was no significant correlation between the refractive error and the change in choroidal thickness from baseline to immediately after warm water immersion (R=-0.058, P=0.710). In addition, there was no significant correlation between the axial length and the change in choroidal thickness from baseline to immediately after warm water immersion (R=0.024, P=0.876).

## Discussion

In this study, choroidal thickness increased significantly along with a significant decrease in SBP, DBP, and MBP values after immersion of the foot in warm water at 40°C for 60 s, with parasympathetic activity being predominant. However, these values returned to their baseline levels within 30 min. There was no significant correlation between the change in choroidal thickness from baseline to immediately after warm water immersion and refractive error and ocular axis length. These results suggest that choroidal changes due to warm water immersion are not affected by refractive error and ocular axis length.

In previous studies of seasonal variation, diurnal variation, and the normal menstrual cycle, choroidal thickness decreases at night, in summer, and during the follicular phase, when parasympathetic activity is predominant [3-5,8]. These findings imply that the predominance of parasympathetic activity leads to vasodilation and an expansion of choroidal volume, potentially increasing choroidal thickness.

A relationship exists between choroidal circulatory dynamics and changes in morphology [13-16]. Nagasato et al. reported that caffeine in coffee causes choroidal vasoconstriction, which consequently increases the choroidal circulatory velocity and thinning of the choroid [16]. Recent studies on normal eyes have shown a significant increase in choroidal circulatory dynamics and a significant decrease in choroidal morphology after a cold pressor test, in which stress increases sympathetic nerve activity [6,7]. In contrast, systemic and choroidal circulatory dynamics decrease significantly after warm water immersion at 40°C for 60 s, which predominates the parasympathetic nervous system [9]; however, the changes in choroidal morphology remain unknown. In this study, when warm water immersion was performed under the same conditions as previously reported, choroidal thickness was significantly increased only immediately after immersion (0 min).

CSC is associated with pathophysiological changes in choroidal circulation and morphology [13,14]. A recent optical coherence tomography angiography (OCTA) report showed characteristics, such as enlargement/distortion of the foveal avascular zone, dark areas (neurosensory detachment/pigment epithelial detachment), dark spots (pigment epithelial detachment), abnormal vessels (dilated vessels/choroidal neovascular membranes), and choriocapillaris island [17]. Reports also indicate that CSC shows increased sympathetic activity, decreased parasympathetic activity, altered sympathetic-parasympathetic balance, and decreased parasympathetic responsiveness [18]. Our findings in this study suggest that increased parasympathetic activity due to thermal stimulation at approximately 40°C may decrease sympathetic activity, even in situations where the sympathetic nervous system is hyperactive.

This study had several limitations. Choroidal thickness was measured manually only in the fovea using a B-scan of EDI-OCT, which may be affected by a measurement bias. To overcome this bias, a binarization evaluation method was used to evaluate the internal structure of the choroid, and automated measurement of a wide range of retinal and choroidal thickness using a C-scan with swept-source OCT should be performed. In addition, as this study followed previously reported sample size and participant recruitment [6,7,9] and exclusively involved immersing both feet in warm water at 40°C for 60 s [9], expanding the sample size and studying variations in choroidal morphology under various thermal stimuli in future research are essential. Furthermore, this study lacks direct measurements of nerve activity, making it challenging to unequivocally attribute the observed changes in choroidal morphology solely to sympathetic or parasympathetic activity. Therefore, evaluation of nerve activity using electrocardiography, alpha-amylase activity assessment in the saliva, and pupillary response assessment should be performed to investigate the relationship with choroidal changes.

## Conclusions

In healthy individuals, parasympathetic activity induced by foot in warm water immersion decreases systemic dynamics and increases the thickness of the choroid. However, the changes were as small as approximately 6 µm, and direct observation of neural activity has not been possible. Future research should directly observe autonomic activity after warm water immersion and assess if enhancing parasympathetic dominance can prevent CSC development and alleviate symptoms related to sympathetic hyperactivity.
